# Supplementary material for: To flow or to grow? Impacts of tapping on sugar maple
Source: Quant Plant Biol. 2025 Apr 7;6:e11. doi: 10.1017/qpb.2025.9 (PMC12035781; doi:10.1017/qpb.2025.9)
Supplement: McNulty et al. supplementary material [file S2632882825000098sup001.docx]

**Supplementary Materials**

Title:

To flow or to grow? Impacts of tapping on sugar maple

Authors:

Hannah McNulty^1^*, Roberto Silvestro^1^, Minhui He^1^, Fabio Gennaretti^2,3^, Sergio Rossi^1^

^1^Laboratoire sur les écosystemes terrestres boreaux, Département des Sciences Fondamentales, Université du Québec à Chicoutimi, Chicoutimi, QC, Canada

^2^ Institut de Recherche sur les Foréts, Groupe de Recherche en Écologie de la MRC-Abitibi, Chaire de Recherche du Canada en dendroécologie et dendroclimatologie, Université du Québec en Abitibi-Témiscamingue, Amos, Canada

^3^ Department of Agricultural, Food and Environmental Sciences, Marche Polytechnic University, Ancona, Italy

* Corresponding author: [hgmcnulty@etu.uqac.ca](mailto:hgmcnulty@etu.uqac.ca) (HM)

**Table S1**. Diameter at breast height (DBH, 1.30m) and total height of the eight monitored trees.

| Tree | Treatment | DBH (cm) | Tree Height (m) |
| --- | --- | --- | --- |
| 1 | Control | 22.9 | 12.7 |
| 2 | Control | 29.1 | 12.1 |
| 3 | Control | 21.6 | 14.3 |
| 4 | Control | 20.9 | 10.8 |
| 5 | Tapped | 33.5 | 14 |
| 6 | Tapped | 21.2 | 13.6 |
| 7 | Tapped | 25.0 | 12.2 |
| 8 | Tapped | 33.8 | 10.5 |

**Table S2.** Effects of year and tapping on tree ring width in sugar maple trees sampled in Simoncouche, Quebec, Canada. The measurements were evaluated by mixed effect models. One and three asterisks indicate P < 0.05 and P < 0.001, respectively.

| Fixed Effect | Estimate | Std. error | t-value |
| --- | --- | --- | --- |
| Intercept | 240.89 | 15.75 | 15.29*** |
| Year 2019-2018 | -71.04 | 12.48 | -5.69*** |
| Year 2020-2019 | -7.86 | 12.48 | -0.63 |
| Year 2021-2020 | 5.07 | 12.48 | 0.41 |
| Tapping | -32.44 | 15.75 | 2.06* |
| Tapping * Year 2019-2018 | -78.09 | 12.48 | -6.26*** |
| Tapping * Year 2020-2019 | 41.26 | 12.48 | 3.31*** |
| Tapping * Year 2021-2020 | -33.80 | 12.48 | -2.71*** |

**Table S3.** Effects of year and tapping on vessel lumen area in sugar maple trees sampled in Simoncouche, Quebec, Canada. The measurements were evaluated by mixed effect models. One and three asterisks indicate P < 0.05 and P < 0.001, respectively.

| Fixed Effect | Estimate | Std. error | t-value |
| --- | --- | --- | --- |
| Intercept | 95.49 | 4.87 | 19.62*** |
| Year 2019-2018 | -0.92 | 4.95 | -0.18 |
| Year 2020-2019 | 2.24 | 5.27 | 0.43 |
| Year 2021-2020 | -0.29 | 5.28 | -0.06 |
| Tapping | 0.87 | 4.87 | 0.18 |
| Tapping * Year 2019-2018 | 8.55 | 4.95 | 1.73 |
| Tapping * Year 2020-2019 | 0.87 | 5.27 | 0.17 |
| Tapping * Year 2021-2020 | -11.20 | 5.28 | -2.11* |

**Table S4.** Effects of year and tapping on vessel density in sugar maple trees sampled in Simoncouche, Quebec, Canada. The measurements were evaluated by mixed effect models. One and three asterisks indicate P < 0.05 and P < 0.001, respectively.

| Fixed Effect | Estimate | Std. error | t-value |
| --- | --- | --- | --- |
| Intercept | 484.99 | 46.71 | 10.38*** |
| Year 2019-2018 | 128.57 | 56.58 | 2.27* |
| Year 2020-2019 | 42.33 | 64.33 | 0.66 |
| Year 2021-2020 | -9.99 | 64.92 | -0.15 |
| Tapping | -52.48 | 46.71 | -1.12 |
| Tapping * Year 2019-2018 | -89.30 | 56.58 | -1.58 |
| Tapping * Year 2020-2019 | 26.62 | 64.33 | 0.41 |
| Tapping * Year 2021-2020 | -10.94 | 64.92 | -0.17 |

**Table S5.** Effects of year and tapping on potential hydraulic conductivity in sugar maple trees sampled in Simoncouche, Quebec, Canada. The measurements were evaluated by mixed effect models. One and three asterisks indicate P < 0.05 and P < 0.001, respectively.

| Fixed Effect | Estimate | Std. error | t-value |
| --- | --- | --- | --- |
| Intercept | 0.07 | 0.009 | 7.45*** |
| Year 2019-2018 | 0.022 | 0.011 | 2.16* |
| Year 2020-2019 | -0.001 | 0.011 | -0.10 |
| Year 2021-2020 | 0.007 | 0.011 | 0.65 |
| Tapping | -0.019 | 0.009 | -2.17* |
| Tapping * Year 2019-2018 | -0.016 | 0.010 | -1.53 |
| Tapping * Year 2020-2019 | 0.014 | 0.011 | 1.32 |
| Tapping * Year 2021-2020 | 0.000094 | 0.011 | 0.01 |

**Table S6.** Effects of year and tapping on hydraulic vessel diameter in sugar maple trees sampled in Simoncouche, Quebec, Canada. The measurements were evaluated by mixed effect models. One and three asterisks indicate P < 0.05 and P < 0.001, respectively.

| Fixed Effect | Estimate | Std. error | t-value |
| --- | --- | --- | --- |
| Intercept | 12.91 | 0.30 | 42.79*** |
| Year 2019-2018 | 0.23 | 0.28 | 0.81 |
| Year 2020-2019 | 0.07 | 0.29 | 0.24 |
| Year 2021-2020 | 0.11 | 0.29 | 0.37 |
| Tapping | 0.35 | 0.30 | 1.17 |
| Tapping * Year 2019-2018 | 0.69 | 0.28 | 2.46* |
| Tapping * Year 2020-2019 | -0.21 | 0.30 | -0.71 |
| Tapping * Year 2021-2020 | -0.50 | 0.30 | -1.68 |


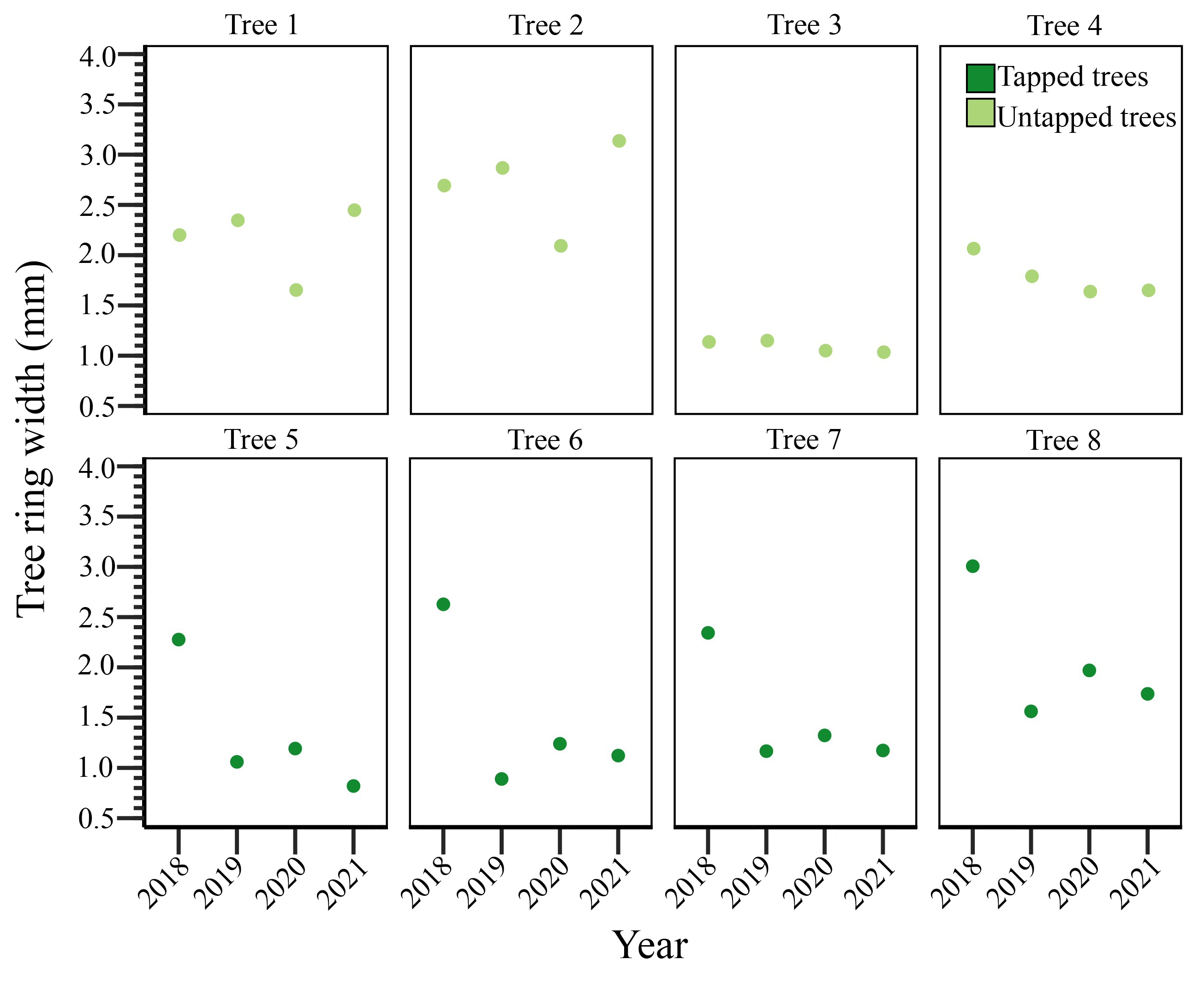


**Figure S1.** Average sugar maple tree ring width by each year, tree, and treatment in Simoncouche, Quebec, Canada


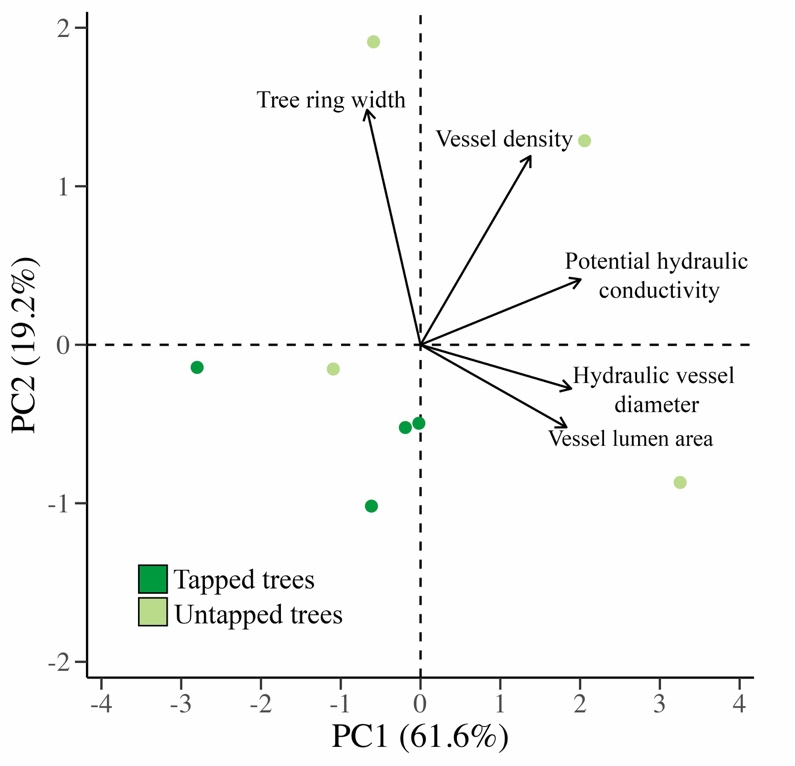


**Figure S2.** Variability between wood anatomical traits in tapped and untapped maples in Simoncouche, Quebec, Canada based on principal component analysis.
